# Complex neurological interplay: adult-onset migraine-triggered seizures in schizencephaly—a case report

**DOI:** 10.3389/fnhum.2025.1606004

**Published:** 2025-10-07

**Authors:** Yuzhu Wang, Fu Zhou, Fei Liu, Xiaohui Wu, Xuan Chen, Zhiqin Xi

**Affiliations:** ^1^Department of Neurology, The People’s Hospital of Dazu, Chongqing, China; ^2^Department of Neurology, The First Affiliated Hospital of Chongqing Medical University, Chongqing, China

**Keywords:** epilepsy, seizures, schizencephaly, migraine, case report

## Abstract

Schizencephaly is a rare congenital cerebral malformation typically diagnosed in early childhood due to serious neurological manifestations. Our case report described a patient with open lip schizencephaly, notable for three unique aspects. First, the patient experienced epileptic seizures in adulthood, which is atypical compared to the usual onset in childhood. Additionally, the patient reported migraine-like pain occurring seconds before each epileptic seizure, with a short interval and regular occurrence. Finally, contrary to the expected severity of symptoms in a patient with open lip schizencephaly, the patient was able to live independently in daily activities, maintained normal cognitive function, and effectively managed both seizures and headaches with standard medication. The purpose of this report is to highlight the significant role of schizencephaly as a causative factor in adult-onset epilepsy and to emphasize the need for a comprehensive therapeutic approach addressing both the structural and functional aspects of this complex neurodevelopmental condition (under the premise of schizencephaly, there were migraine-like attacks in the short term before epileptic seizures).

## Introduction

Schizencephaly is a rare congenital malformation of the central nervous system characterized by a cleft extending from the cerebral hemispheres to the lateral ventricles, lined with heterotopic gray matter (Halabuda et al., 2015). This disorder is thought to result from a localized failure in the induction of neuronal migration from the ventricular zone (Barkovich and Kjos, 1992). Based on cleft openness and affected regions, clefts are classified into two subtypes: Type I, with closed lips not connecting to the lateral ventricle, and Type II, with open lips linking the subpial space to the lateral ventricle (Braga et al., 2018). Epilepsy is a common and often severe neurological manifestation in patients with schizencephaly, with most experiencing seizures before the age of three, which frequently leads to the initial diagnosis during medical evaluation (Guerrini and Carrozzo, 2002).

Although the comorbidity of epilepsy and migraine is not uncommon, the occurrence of brief migraine-like attacks immediately preceding epileptic seizures is rare in patients with schizencephaly. Here, we report a 26-year-old female with schizencephaly who presented with adult-onset epilepsy accompanied by migraine-like episodes preceding seizures, highlighting a unique clinical presentation and the effectiveness of standard therapeutic intervention.

## Case presentation

**FIGURE 1:**
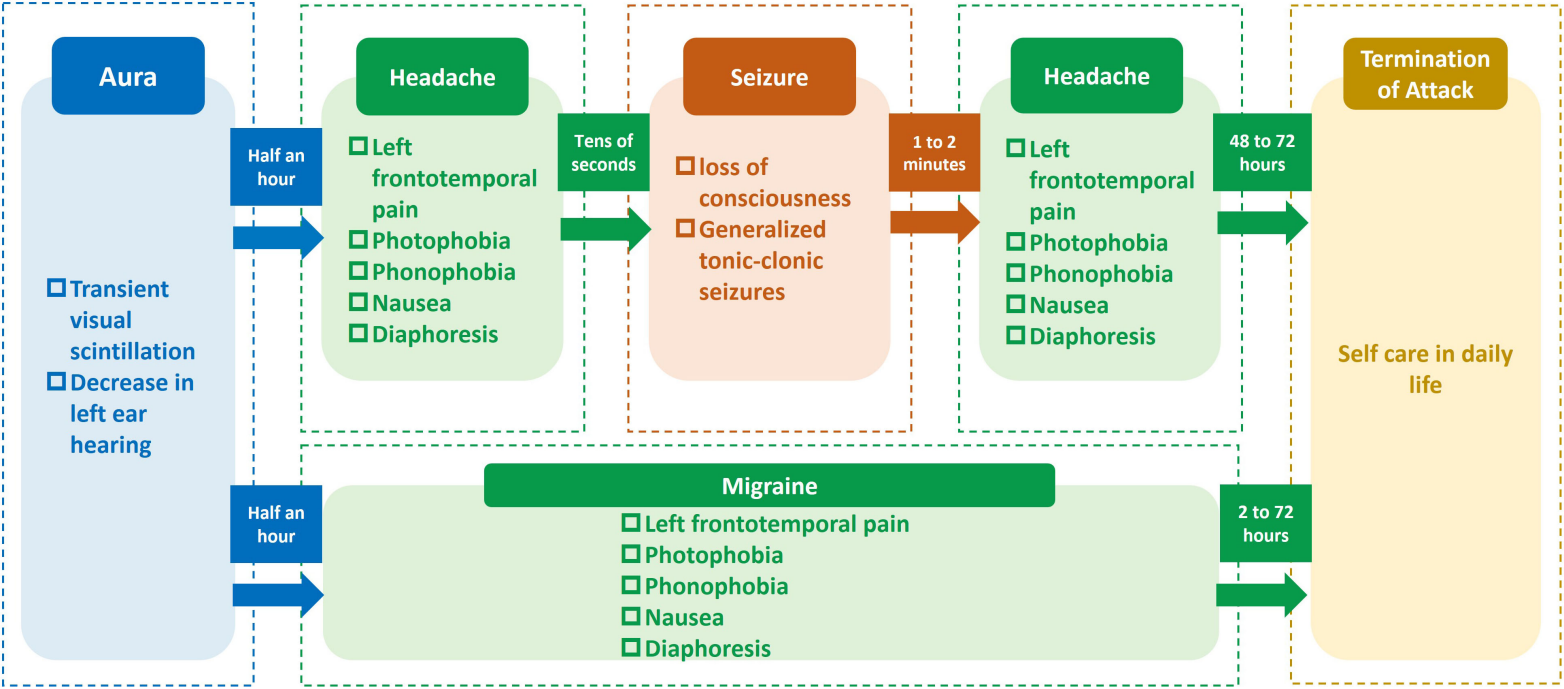
Schematic diagram of the process of migraine and epileptic seizures in the schizencephaly patient.

A 26-year-old female presented for evaluation of seizures and headaches. She was born at term without perinatal complications, and her family, medical, and surgical histories were unremarkable. The patient has a documented history of epileptic seizures over the past 8 years, manifested as generalized tonic-clonic seizures. Concurrently, since 8 years ago, the patient has experienced headache episodes, typically presenting with transient visual scintillations and a nearly half-hour decrease in left ear hearing followed by left frontotemporal distension pain, sensations of head heaviness, photophobia, phonophobia, nausea, and sweating, which required rest for relief. These headache episodes lasted 2–72 h and occurred 4–5 times per month, with clinical features meeting the diagnostic criteria for migraine with aura (Eigenbrodt et al., 2021), established according to the International Classification of Headache Disorders, 3rd edition (ICHD-3) (Headache Classification Committee of the International Headache Society [IHS], 2013). Notably, each epileptic seizure was preceded by a brief headache episode, separated by only decades of seconds, with the pre-seizure headaches mirroring her regular migraine in aura, pain characteristics, location, and accompanying symptoms. During the interictal period of seizures, the patient remained alert but experienced transient amnesia, with the continuation of the headache above symptoms persisting for 48–72 h. A diagram of the symptom progression is shown in Figure 1. Over the past 8 years, the patient has been treated solely with sodium valproate for epilepsy, with no seizures occurring during regular medication adherence. However, the patient has discontinued medication twice on her own, each cessation resulting in seizure recurrence.

**FIGURE 2:**
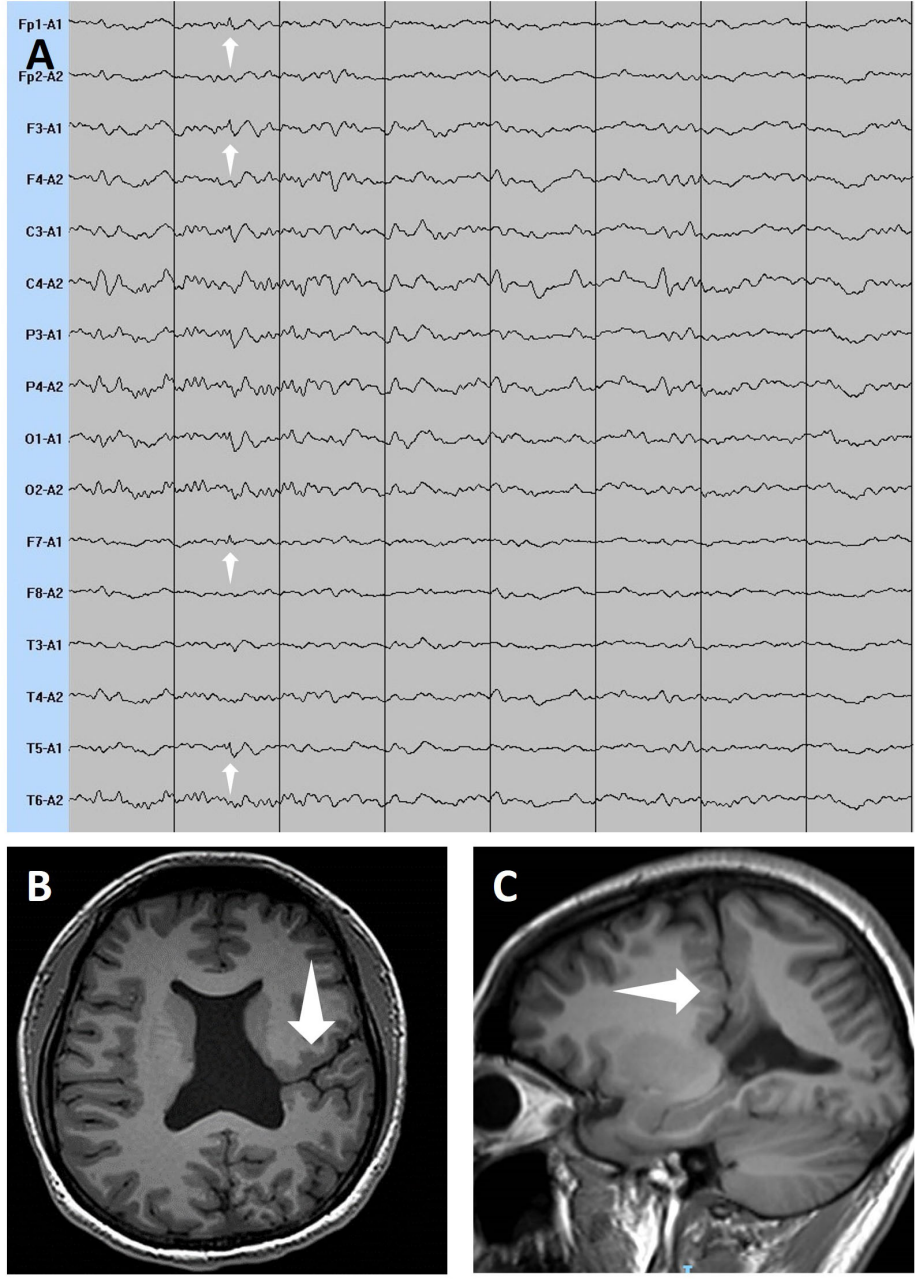
The white arrows in the electroencephalogram **(A)** show low to medium-amplitude spike waves in the left frontal pole, dorsolateral prefrontal cortex, and posterior temporal gyrus. The white arrows in the axial view **(B)** and sagittal view **(C)** of T1-weighted magnetic resonance imaging show a cleft extending from the subarachnoid space of the left frontal lobe region to the left lateral ventricle, filled with cerebrospinal fluid.

The patient has a height of 159 cm and a weight of 59 kg, with no evidence of developmental delays. Neurological examination revealed decreased muscle strength (Grade 4/5) in the right limbs, with no other positive neurological signs. When the patient reported severe headaches, the electroencephalogram (EEG) showed no continuous epileptiform discharges. However, paroxysmal low- to medium-amplitude spike waves were observed in the left brain, although no ictal events were captured during the 3-day continuous EEG monitoring after admission. Non-contrast cranial magnetic resonance imaging confirmed an open lip schizencephaly in the left frontal lobe (Figure 2). Cranial Doppler ultrasound showed no significant abnormalities. Given that the location of the patient’s malformation coincided with the area of epileptiform discharges indicated, and considering the absence of head trauma, cerebrovascular disease, genetic factors, or infections as other causes of epilepsy (Thijs et al., 2019) the seizures were supposed to be secondary to schizencephaly. No abnormalities were found in the right heart echocardiography, ruling out the possibility of migraines secondary to a patent foramen ovale (West et al., 2018). The Mini-Mental State Examination (MMSE) score exhibited no significant neurocognitive dysfunction. Finally, the patient was diagnosed with schizencephaly, epilepsy, and migraine with aura. Treatment was adjusted to ibuprofen for headache relief and levetiracetam for epilepsy management. Before treatment adjustment, the patient experienced 4–5 migraine attacks per month, often accompanied by seizures when antiepileptic medication was discontinued. Treatment was then adjusted to levetiracetam for epilepsy management, and ibuprofen was prescribed only for acute headache relief. Ibuprofen was taken for approximately 1 week to control residual headache symptoms and was then discontinued when no further headaches occurred. During the subsequent 3-month follow-up, the patient remained free of both seizures and migraine attacks without further need for ibuprofen.

## Discussion

Our case presented a unilateral open lip schizencephaly. Open lip clefts typically lead to more severe impairment, such as epileptic seizures that most patients experience starting before the age of three (Kamble et al., 2017). However, our patient exhibited a later onset of epilepsy, having never experienced seizures until adulthood. At present, only four cases of adult-onset epilepsy with schizencephaly have been reported (Papayannis et al., 2012; Kamble et al., 2017; Battah et al., 2022; Laasri et al., 2023). We found that adult-onset epilepsy patients with schizencephaly seem to have milder clinical symptoms than those that occurred epilepsy in childhood, such as no obvious developmental delay or neurocognitive dysfunction, leading to a lack of willingness to seek early medical attention. Our patients have no significant differences in appearance and intelligence compared to normal peers during the interval of epileptic seizures and are generally self-sufficient in daily life.

Based on clinical presentation, our patient fulfilled the ICHD-3 diagnostic criteria for migraine with aura. Although autonomic symptoms such as nausea and sweating can overlap with ictal prodromes, several features strongly supported a migraine diagnosis: recurrent and stereotyped attacks over 8 years, consistent visual aura and photophobia, and prolonged headache duration of 2–72 h (Headache Classification Committee of the International Headache Society [IHS], 2013). Moreover, features typically associated with secondary headaches from structural brain lesions—such as progression, persistence, or signs of elevated intracranial pressure or focal deficits—were absent (Zhu et al., 2020; Robbins, 2021). These characteristics further support the interpretation of primary migraine rather than secondary headache due to schizencephaly. In addition to typical migraine attacks, the patient also experienced headaches decades of seconds before each epileptic seizure. Due to the brief duration of these pre-seizure headaches, we were initially unsure whether they were focal epileptic manifestations or independent headaches. However, repeated history taking by two neurologists confirmed that the headaches occurring interictally, pre-ictally, and post-ictally were identical in quality, intensity, and location, all fulfilling ICHD-3 criteria. Together with the absence of continuous ictal discharges during EEG monitoring and the favorable response to combined therapy, we favored the interpretation that the seizures were triggered by migraine attacks. Comorbidity of migraine and epilepsy is not uncommon (Keezer et al., 2016). Our case therefore highlights the complex interaction between structural brain anomalies, migraine, and epileptogenesis.

Additionally, statistical analysis of 734 patients with schizencephaly demonstrated that patients with open lip clefts had more treatment-resistant seizures (54.0%) compared to patients with closed lip clefts (15.0%) (Braga et al., 2018). However, over the past 8 years, regular use of sodium valproate has led to the cessation of epileptic seizures. Recurrent epileptic seizures are attributed to self-discontinuation of medication. Kanner et al. pointed out that the presence of comorbidities and the concurrent use of medication should be considered when choosing antiepileptic drugs (Kanner and Bicchi, 2022). Our patients showed better efficacy in combination with anti-migraine drugs compared to using anti-epileptic drugs alone. This case has the limitation that ictal video-EEG data were not available. Although the patient underwent continuous EEG monitoring for 3 days during hospitalization, no seizures or epileptiform discharges were observed after initiation of combined antiseizure and antimigraine therapy. Thus, direct electrophysiological confirmation of the pre-seizure headaches was not possible. Nevertheless, the long-standing, stereotyped clinical features and the favorable therapeutic response supported the interpretation of migraine with aura.

## Conclusion

In summary, our case highlights an unusual presentation of open lip schizencephaly. The case underscores the need to raise awareness of schizencephaly as a serious cause of adult-onset epilepsy. Simultaneously, the successively occurring of migraines and seizures in patients with schizencephaly within a short period of time may reflect a shared pathophysiological substrate, which emphasizes the importance for integrated therapeutic approaches targeting both the structural and functional aspects of this complex neurodevelopmental condition.

## Data Availability

The original contributions presented in this study are included in this article/supplementary material, further inquiries can be directed to the corresponding author.
